# An NIR photothermal-responsive hybrid hydrogel for enhanced wound healing

**DOI:** 10.1016/j.bioactmat.2022.03.006

**Published:** 2022-03-10

**Authors:** Lin Jin, Xiaoqing Guo, Di Gao, Yan Liu, Jiahua Ni, Zhiming Zhang, Yiqiao Huang, Guibin Xu, Zhe Yang, Xingcai Zhang, Xianhan Jiang

**Affiliations:** a Department of Urology, The Fifth Affiliated Hospital of Guangzhou Medical University, Guangzhou, 510700, PR China; b International Joint Research Laboratory for Biomedical Nanomaterials of Henan, Zhoukou Normal University, Zhoukou, 466001, PR China; c The Key Laboratory of Biomedical Information Engineering of Ministry of Education, School of Life Science and Technology, Xi'an Jiaotong University, Xi'an, 710049, PR China; d School of Engineering and Applied Sciences, Harvard University, Cambridge, MA, 02138, USA; e School of Engineering, Massachusetts Institute of Technology, Cambridge, MA, 02139, USA; f Research Institute of Xi'an Jiaotong University, Hangzhou, Zhejiang, 311200, PR China

**Keywords:** Angiogenesis promotion, NIR response, Hydrogel, Nanofibers, Scarless wound healing

## Abstract

Moderately regulating vascularization and immune microenvironment of wound site is necessary to achieve scarless wound healing of the skin. Herein, we have prepared an angiogenesis-promoting and scar-preventing band-aid with a core-shell structure, that consists of MXene-loaded nanofibers (MNFs) as the core and dopamine-hyaluronic acid hydrogel (H) as the shell (MNFs@V–H@DA) to encapsulate a growth factor (vascular endothelial growth factor, VEGF, abbreviated as V) and H_2_S donor (diallyl trisulfide, DATS, abbreviated as DA). The continuous release of DA from this system produced H_2_S, which would successfully induce macrophages to polarize into M2-lile phenotype, regulating the immune microenvironment and inhibiting an excessive inflammatory response at the wound sites. It is conducive to the proliferation of skin cells, facilitating the wound healing. In addition, an appropriate amount of VEGF can be released from the MXene nanofibrous skeleton by adjusting the time of near-infrared (NIR) light exposure, preventing excessive neovascularization and extracellular matrix deposition at the wound sites. Collectively, this NIR photothermal-responsive band-aid achieved scarless wound healing through gradient-controlled vascularization and a related immune sequential reaction of damaged skin tissue.

## Highlights

• A hybrid system combining hydrogel and nanofibers developed as band-aid for wound healing.
• Mild hyperthermia induced by photothermal therapy achieves VEGF controlled release for appropriate neovascularization of wound site.
• Continuous release of H_2_S facilitates the polarization of macrophages toward M2-like phenotype to regulate immune microenvironment of wound site.
• The synergism of neovascularization and immune regulation accelerates the wound healing.

## Graphical abstract

An angiogenesis-promoting and scar-preventing band-aid that consists of MXene-loaed nanofibers (MNFs) as the core and dopamine-hydroxyapatite hydrogel (H) as the shell (MNFs@V–H@DA) was prepared. This NIR photothermal-responsive band-aid achieved scarless wound healing through gradient-controlled vascularization and a related immune sequential reaction of damaged skin tissue, which is a promising strategy to achieve scarless wound healing.

**Figure:**
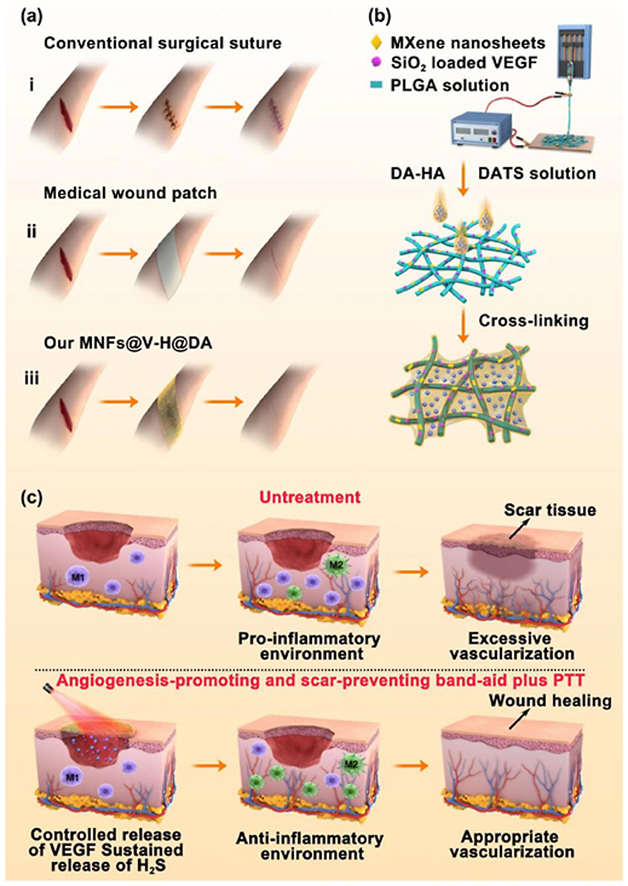

## Abbreviations

VEGF: vascular endothelial growth factor
DA: diallyl trisulfide
MNFs: MXene-loaed nanofibers
MNFs@V: MXene nanofibers @ VEGF-loaded SiO_2_ NPs
H@DA: dopamine-hyaluronic acid hydrogel contain DATS
NIR: near-infrared
MNFs@V–H@DA: MXene nanofibers @ VEGF core with dopamine-hyaluronic acid hydrogel@DATS shell
PLGA: poly(lactide-co-glycolide)
NFs: PLGA nanofibers
MSCs: mesenchymal stem cell
TCPs: Tissue culture plates
NPs: Nanoparticles
SEM: scanning electron microscope
TEM: transmission electronic microscope

## 1 Introduction

Fibrotic scar tissue formation is a common problem in skin tissue repair [[1], [2], [3], [4]]. These scar sites have many disadvantages compared with normal skin tissue, and lots of functions are lost [[5], [6], [7], [8]]. For example, dermal appendages may be lost, and the scarred skin exhibits a fibrotic extracellular matrix (ECM) with dense, parallel fibers, which greatly affected the flexibility and strength of skin [[9], [10], [11]]. This alters the fiber structure and makes the scar tissue weaker than unwounded skin [12,13]. Consequently, achieving scarless wound healing has become an important clinical goal.

Neovascularization provides necessary nutrients for the proliferation and differentiation of new cells at the wound site in the process of wound healing [[14], [15], [16]]. An appropriate amount of neovascularization is necessary to repair the damaged tissue and is beneficial to wound healing, however, excessive vascularization produces a series of side effects, aggravates the deterioration of wound tissues, and leads to the formation of scars [[17], [18], [19]]. At the same time, inflammation is one of the most basic response to injury in the body [[20], [21], [22]]. Keratinocytes and fibroblasts produce various cytokines involved in the inflammatory response and in regulating immune function under a stimulated state [1,23]. Appropriate inflammation can promote wound healing, however, an excessive inflammatory response also can cause significant damage [[24], [25], [26]]. Therefore, rapid anti-inflammatory therapy in the early stage of skin repair could prevent the enrichment of local inflammatory cells, inhibit the storm of inflammatory cytokines, and prevent the aggravation of wound damage [27,28]. More importantly, the repair-promoting function of anti-inflammatory M_2_ macrophages can inhibit the excessive proliferation of fibroblasts and the excessive deposition of the extracellular matrix in the late stage of wound healing, thus achieving scarless skin healing [29,30]. Consequently, achieving appropriate neovascularization and regulating the immune microenvironment at the wound site are the key points for rapid skin repair and scarless healing.

Various biomaterials have been used for skin tissue regeneration and repair, such as hydrogels [31,32], nanofibers [14,33]. However, the practical application of these biomaterials depends on their ability to meet the complicated functional requirements of the injured tissue. How to achieve the programmed release of different drugs through different scaffolds is an important issue. Fibers and hydrogel materials have their own unique advantages [[34], [35], [36], [37]]. The release performance of fibrous materials as drug carriers is often affected by swelling of the fiber interface. Meanwhile hydrogels as drug vectors can more easily exchange with cells or tissue due to their sufficient water content. Therefore, a fiber material loaded with positive factors can realize controllable release by swelling of the interface. On the other hand, a hydrogel system can also regulate the exchange rate with the cells or tissue by microenvironment. Therefore, the precise release of neovascularization and anti-inflammatory factors can be achieved through the fibers and hydrogel double structure system as carriers.

As a new two-dimensional transition metal material, MXene with unique chemical and physical properties [14,38], especially high photothermal conversion efficiency attracted biomaterials researches to expand its biomedical applications [39,40]. In this study, based on MXene, we have developed a NIR-responsive band-aid (MNFs@V–H@DA, that is, MXene nanofibers @ VEGF core with dopamine-hyaluronic acid hydrogel@DA shell), combining advantages of nanofibers and hydrogels [33,41,42]. The results indicated that the obtained MNFs@V–H@DA with suitable NIR laser irradiation condition promoted vascularization and regulated the immune microenvironment at the wound site to achieve better wound healing, which effectively inhibited the excessive proliferation of repair cells, such as epidermal cells, vascular endothelial cells, and prevented the excessive secretion and deposition of extracellular matrix proteins. Moreover, it also promoted fibroblast migration and achieved scarless wound healing.

## 2 Results and discussion

### 2.1 Fabrication and characterization of the MNFs@V–H@DA platform

**Fig. 1:**
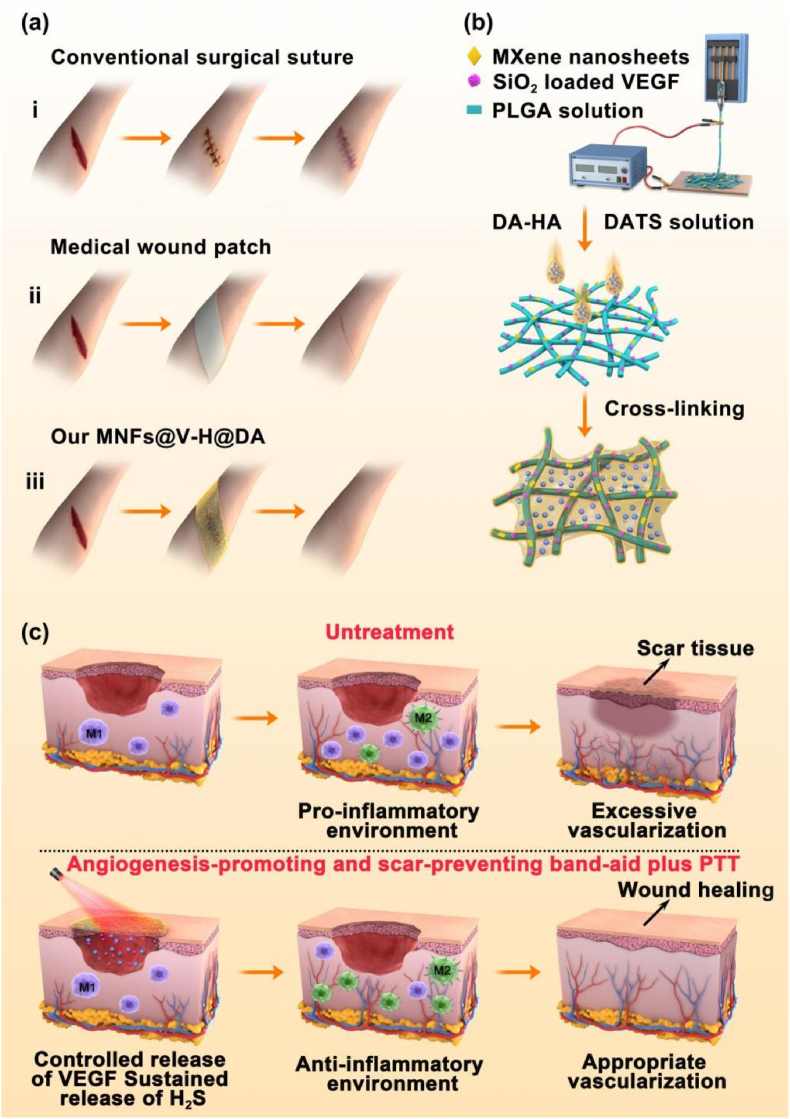
Preparation process, and wound healing process of MNFs@V–H@DA. Various wound healing materials (a), i: traditional surgical suture, ii: medical wound patch, iii: our MNFs@V–H@DA. Scheme of MNFs@V–H@DA fabrication by electrospinning process and surface coating (b). The mechanism of MNFs@V–H@DA on wound healing (c).

The traditional surgical suture (Fig. 1ai) and current medical wound patch (Fig. 1aii) that still leaves scars after treatment, which affect the effect of wound healing and recovery of skin function. In this study, we prepared MNFs@V–H@DA as Fig. 1b, which with suitable NIR laser irradiation promoted vascularization and regulated the immune microenvironment at the wound site to achieve scarless wound healing (Fig. 1aiii). The key strategy in the design of MNFs@V–H@DA is that the MXene and VEGF-loaed PLGA nanofibrous skeleton could heat the wound site under NIR irradiation as well as achieve appropriate release of VEGF through “on/off” of NIR irradiation. Meanwhile, the hydrogel would achieve continuous release of DA, and which produced H_2_S and promoted polarization of macrophages from pro-inflammatory M_1_ macrophages to anti-inflammatory M_2_ macrophages (Fig. 1c). In addition, the H_2_S increased the anti-inflammatory factor IL4 and reduced the pro-inflammatory factors IFN-γ and TNF-α, finally regulating the immune microenvironment at the wound site.

**Fig. 2:**
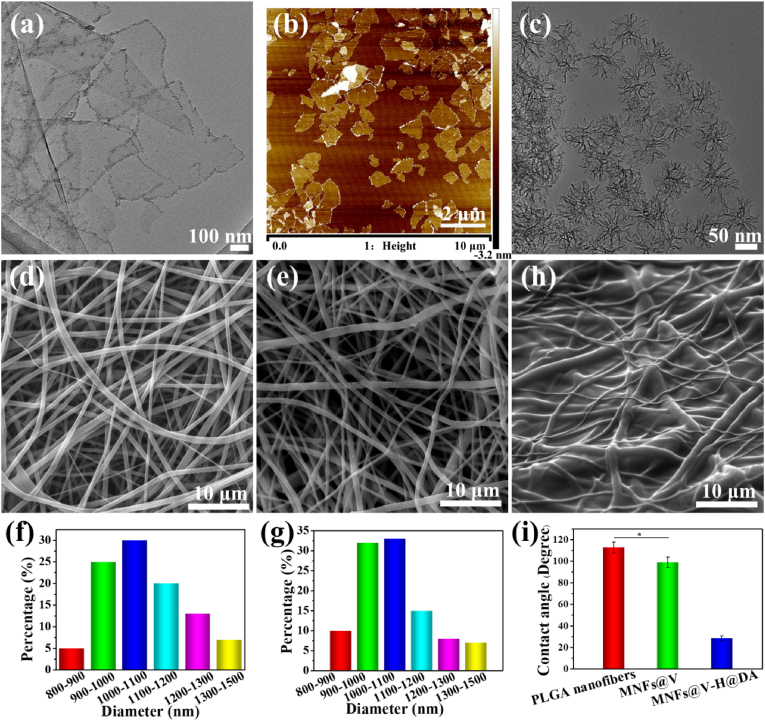
Morphology characterization of MXene nanosheets, SiO_2_ NPs and MNFs@V–H@DA. TEM and AFM images of MXene nanosheets (a, b). TEM image of SiO_2_ NPs (c). SEM images of PLGA (d), MNFs@V (e), MNFs@V–H@DA (f). Diameter distribution of PLGA (f) and MNFs@V (g). The SEM image of MNFs@V–H@DA (h). The contact angels of PLGA nanofibers, MNFs@V, MNFs@V–H@DA (i). Data are presented as mean ± standard deviation, n = 3, *p value < 0.05.

The band-aid in this study consisted of the PLGA nanofibers as the core that encapsulated MXene nanosheets and VEGF-loaded mesoporous silica nanoparticles (SiO_2_ NPs) and dopamine-hyaluronic acid (HA) hydrogels as shell that encapsulate the resultant fibers and H_2_S donor DAST. Thus, the different components in our system were prepared and characterized, firstly. As shown in Fig. 2a and b, MXene nanosheets clearly showed the presence of a single layer and confirmed the size distribution of 300–500 nm. To ensure that VEGF was fully dispersed and released in an orderly and controllable manner, VEGF was loaded into the pores of SiO_2_ nanoparticles (NPs, with a diameter of approximately 70 nm), as shown by TEM (Fig. 2c) imaging. Furthermore, the pore diameter distribution indicated that SiO_2_ NPs have a pore size of 16 nm (Fig. S1), which ensured that VEGF could enter easily into the pores.

Then, MXene nanosheets and VEGF-loaded SiO_2_ NPs were mixed with poly(lactide-co-glycolide) (PLGA) solutions to prepare nanofibers, and fabricated using an electrospinning process, which form functional skeletons and provide a source of photothermal properties. The scanning electron microscope (SEM) images of PLGA nanofibers (Fig. 2d) and MNFs@V (MXene nanofibers @ VEGF-loaded SiO_2_ NPs) (Fig. 2e) indicated that the surface of the nanofibers in the MNFs@V appeared the same as unmodified PLGA nanofibers, with no apparent change after doping with MXene nanosheets and VEGF-loaded SiO_2_ NPs. The only change was in the diameter distribution, which was more concentrated in the range of 900–1100 nm (Fig. 2f and g). Next, a D-H (dopamine-hyaluronic acid conjugate) aqueous solution containing DA was spin-coated on the above mentioned MNFs@V nanofibers. After 6 h oxidative polymerization, nanofibers were successfully coated with the dopamine-hyaluronic acid hydrogel (H). Therefore, MNFs@V–H@DA was successfully prepared. SEM imaging indicated that the hydrogel layer was well distributed on the nanofibers and the nanofibrous pattern was visible in the MNFs@V–H@DA (Fig. 2h). As shown in Fig. S2, the thickness of the MNFs@V–H@DA was approximately 30 μm. Moreover, the contact angles of PLGA nanofibers, MNFs@V, MNFs@V–H@DA (Fig. 2i) indicated that the hydrophilicity gradually increased after doping with the MXene nanosheets and hydrogel layer. Initially, the contact angle of PLGA nanofibers was 112.7°. After doping with MXene nanosheets and VEGF-loaded SiO_2_ NPs, the contact angle decreased to 99.0°, whereas after coating with a dopamine-hyaluronic acid hydrogel layer, the contact angle was reduced to only 28.6°. This indicated that the hydrophilic property of the MNFs@V–H@DA was greatly improved, confirming that MNFs@V–H@DA could make good contact with the wound area and provide a beneficial microenvironment, allowing positive factors to diffuse into the wound tissue, and ultimately achieving the desired state for wound healing.

The mapping pattern was characterized for chemical characterization of MNFs@V. (Fig. S3), which clearly showed the constituent elements of doped MXene nanosheets and SiO_2_ NPs, such as C, O, Ti, and Si. In addition, the X-ray diffraction pattern also showed prominent characteristic peaks of MXene and SiO_2_ NPs (Fig. S4). These results indicated that MXene nanosheets and SiO_2_ NPs were successfully embedded into the nanofibers.

### 2.2 Photothermal conversion of MNFs@V–H@DA

**Fig. 3:**
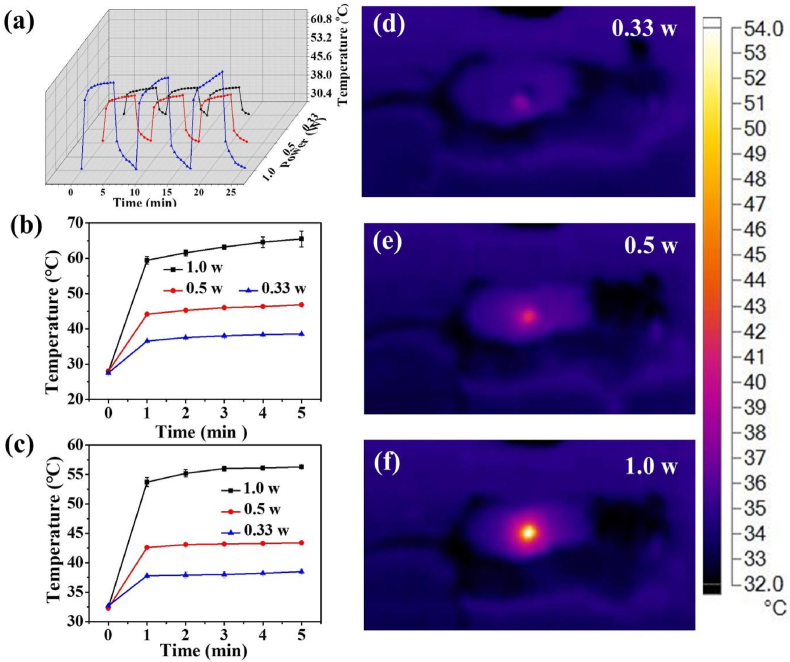
Photothermal property of MNFs@V–H@DA. Photothermal conversion of MNFs@V and MNFs@V–H@DA. Temperature rising profiles of the MNFs@V under NIR light of 0.33 W, 0.50 W and 1.0 W during 3 on/off cycles (a). Temperature changes of M-NFs@V in aqueous solution (b) and MNFs@V–H@DA on the wound area (c). Thermal images of MNFs@V–H@DA on the wound area (d, e, and f) under NIR light of 0.33 W/cm^2^, 0.50 W/cm^2^ and 1.0 W/cm^2^.

To obtain a NIR-responsive nanofiber-hydrogel band-aid, MXene nanosheets as a photothermal source were introduced into the nanofibers. Under NIR irradiation, the obtained MNFs@V demonstrated excellent photothermal performance. For example, the repeated heating behavior of MNFs@V upon NIR irradiation was tested over three consecutive on/off cycles after NIR exposure. For each cycle, the obtained M-NFs@V was heated up to 38.6 °C at 0.33 W/cm^2^ (Fig. 3a); when the NIR laser was switched off, it immediately cooled down to the initial temperature. In addition, at power levels of 0.5 W/cm^2^ and 1.0 W/cm^2^, the system also displayed good heating stability. Furthermore, when nanofibers were exposed to NIR laser at a power density of 0.33 W/cm^2^, 0.5 W/cm^2^, and 1.0 W/cm^2^ for 5 min, the temperature increased to 38.6 °C, 46.7 °C, and 63.3 °C, respectively (Fig. 3b). Thermal images of MNFs@V under NIR laser irradiation, shown in Fig. S5, demonstrate a similar phenomenon, further confirming the heating capacity of this system. Notably, after coating with D-H hydrogel, the photothermal property of MNFs@V–H@DA was still retained, and the system was heated to 38.5 °C upon laser irradiation at 0.33 W/cm^2^ (Fig. 3c). Furthermore, when the NIR laser power was increased to 0.5 W/cm^2^ and 1.0 W/cm^2^, the solution was heated to 43.4 °C and 56.3 °C, demonstrating the better photothermal behavior of the nanofibers.

Considering that high temperatures can be harmful to the surrounding skin and 0.33 W/cm^2^ is used in clinical therapy, 0.33 W/cm^2^ was chosen for the *in vivo* investigation. As shown in Fig. 3d, e, and f, the MNFs@V–H@DA also displayed ideal photothermal conversion when used on mouse dorsal skin. Furthermore, thermal images indicated that the local temperature of the MNFs@V–H@DA model area increased to 38.5 °C after 5 min of laser irradiation (Fig. S6). These results indicated that the MNFs@V–H@DA also had desirable heating performance *in vivo*.

### 2.3 Biocompatibility of MNFs@V–H@DA platform *in vitro*

**Fig. 4:**
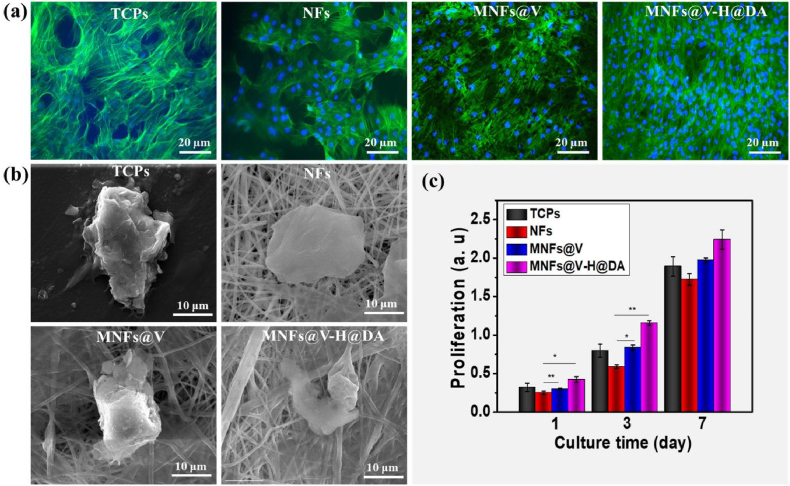
Biocompatibility of MNFs@V–H@DA. Fluorescent images (a) and cellular morphology (b) of MSCs cultured 5 days on TCPs, PLGA nanofibers, MNFs@V and MNFs@V–H@DA. The cellular proliferation (c) of MSCs cultured on TCPs, NFs, MNFs@V and MNFs@V–H@DA with 7 days period. Data are presented as mean ± standard deviation, n = 3, *p value < 0.05, **p value < 0.01.

To evaluate the biocompatibility of the MNFs@V–H@DA, the cellular cytotoxicity, activity and morphology were evaluated. Calcein AM staining (Fig. 4a) of cells cultured on the MNFs@V–H@DA, MNFs@V, NFs and TCPs indicated that MSCs displayed excellent cellular activity and grew along the fibrous pattern. In addition, the number of cells on the MNFs@V–H@DA was much higher than that on the MNFs@V, NFs and TCPs, which was consistent with the results of the cell proliferation study.

Examination of cell morphology also demonstrated that MSCs on the MNFs@V–H@DA spread widely along the pattern of fibers and even formed integrated cell-fiber constructs (Fig. 4b). This was attributed to VEGF release and the presence of hydroxyl, and amino groups contained in the dopamine-hyaluronic acid hydrogel, providing the desired microenvironment for cell growth. Moreover, we also found that the surface of MNFs@V–H@DA was slightly changed because of the uptake of VEGF and MXene nanosheets by MSCs. In contrast, cells on the NFs demonstrated a significant contraction, whereas cells on the MNFs@V displayed slightly better extension along the nanofibers and better contact with the nanofibrous surface compared to that on the PLGA nanofibers. These results indicated that MNFs@V–H@DA provided the required elements or functional groups for cellular growth and proliferation while also supplying spatial structure for cells to achieve excellent cellular activity.

The cellular cytotoxicity of the MNFs@V–H@DA and MNFs@V was tested by the CCK-8 assay, TCPs and NFs were used as the control groups. MSCs were cultured on samples and incubated for evaluation. The results revealed that cells on the MNFs@V–H@DA exhibited much better cell attachment and proliferation at the various time-points than those on the two control groups during the culture period (Fig. 4c), indicating that it provided an excellent environment for cell attachment and growth. Moreover, cells on the MNFs@V showed much better cell proliferation than those on the PLGA nanofibers, it displayed a same level of cells on the TCPs after 7 days cultured, which indicated that MXene with positive groups and VEGF improved cell growth. These test results demonstrated that the prepared MNFs@V–H@DA had excellent biocompatibility and was suitable for use to promote scarless wound healing.

Based on the excellent photothermal performance of MNFs@V–H@DA, the MXene-PLGA composite nanofibers forming the skeleton displayed a swollen state because of the glass transition temperature of PLGA under NIR exposure. Then, VEGF released from PLGA nanofibers, especially stable and controlled VEGF release through on/off NIR irradiation (Fig. S7a), enhanced suitable neovascularization and promoted wound healing. More importantly, at the same time, DATS dispersed in hydrogels also was also released in a sustained way (Fig. S7b). No significant difference was observed under the condition on/off NIR, which produced H_2_S to regulate the immune microenvironment at the wound site. Thus, the synergistic effect of the two factors could effectively promote wound healing.

### 2.4 Evaluation of MNFs@V–H@DA system for wound healing *in vivo*

To evaluate the feasibility of therapeutic use of MNFs@V–H@DA, intermittent NIR irradiation was used for animal experiments, and the effective irradiation period of the MNFs@V–H@DA to achieve adequate availability of VEGF and DATS was 3 min. After NIR irradiation, some pores could be observed by SEM image (Fig. S8) due to PLGA swelling or glass transition state, which indicated that PLGA underwent phase change at high temperatures. To evaluate the efficacy of MNFs@V–H@DA on wound healing, a mouse wound-healing model was established, which involved creating circular wounds 0.8 cm in diameter on the backs of mice, which were treated with MNFs@V–H@DA with NIR laser irradiation for 7 or 14 days, MNFs@V–H@DA without NIR laser irradiation, or phosphate-buffered saline (PBS). Thermal imaging of mice treated with MNFs@V–H@DA with NIR laser irradiation at 0.33 W/cm^2^ indicated that the temperature also increased quickly under NIR exposure. The healing condition of the wound areas was observed at fixed time-points and analyzed.

**Fig. 5:**
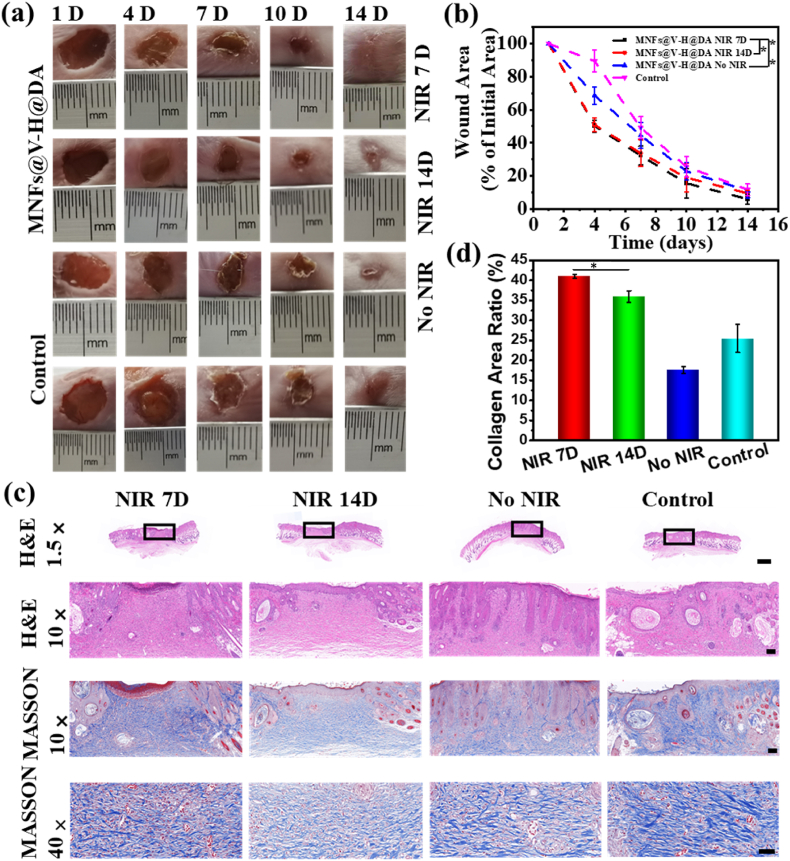
Evaluation of the MNFs@V–H@DA with various conditions on wound healing of rats. Representative photo images (a) and the wound area (b) of various groups of the skin wounds of various groups (control group, MNFs@V–H@DA, MNFs@V–H@DA + NIR 7 days group, MNFs@V–H@DA+14 days NIR group) on day 1, 4, 7, 10, 14. The scale bars are 1.0 cm, data are presented as mean ± standard deviation, n = 5, *p value < 0.05, **p value < 0.01. (c) Corresponding H&E and masson staining of the wound beds on day 14. The scale bars are 1000, 100, 100, and 20 μm, respectively. (d) Quantitative analysis evaluation of area masson, data are presented as mean ± standard deviation, n = 3, *p value < 0.05.

The MNFs@V–H@DA 7-day and 14-day NIR laser irradiation groups displayed much better recovery than the group without NIR laser irradiation and the control group. The wounds treated with MNFs@V–H@DA with NIR laser irradiation for 7 days showed an obvious response and good healed effect on day 14 (Fig. 5a), which was much better than the group that underwent 14 days of NIR laser irradiation. More importantly, the scarring of the animals with 7 days of NIR exposure was significantly less than those in the 14-day NIR exposure group. Measurement of the wound area also indicated a similar result to the wound images (Fig. 5b), the wound area of MNFs@V–H@DA 7-day NIR laser irradiation groups was less 14.05% compared to that 7-day NIR laser irradiation groups. In addition, the condition of the wound beds in terms of constriction and epithelization was recorded and analyzed using hematoxylin-eosin (H&E) staining. The results indicated that the wounds in the MNFs@V–H@DA with NIR laser irradiation group contracted faster, and the new epithelial tissues were much thicker than in the other groups (Fig. 5c). Meanwhile, the scarless phenomenon was much better than that in the group exposed to NIR laser irradiation for 14 days, which indicated that appropriate NIR laser irradiation provided ideal healing conditions due to the appropriate amounts of VEGF released from MNFs@V–H@DA.

Additionally, Masson's staining also showed that the MNFs@V–H@DA + 7 days of NIR irradiation group formed a better tissue structure compared to other groups. Fig. 5c and d exhibit that the expression of Masson after the treatment of MNFs@V–H@DA without NIR irradiation is lower than control group. This phenomenon may be caused by the following two factors. On one aspect, several previous studies have demonstrated that mild hyperthermia is beneficial for wound healing. Meanwhile, due to the lack of hyperthermia, the VEGF in PLGA nanofibers is hard to release without exogenous stimuli, which slows down the vascularization at wound site, suppressing the nutrient substances transportation and the wound healing. On another aspect, without above advantages for wound healing, the band-aid may hinder the closure of cells at the edge of wound site, thus, there is no significant enhancement on wound healing and lower Masson expression in the group of MNFs@V–H@DA without NIR irradiation compared to control group.

Statistical analysis was used to further evaluate the therapeutic effects of various treatments, suggesting that the MNFs@V–H@DA without NIR group displayed worse recovery than the MNFs@V–H@DA with NIR groups. These results may be attributable to the following factors: 1) Photothermal therapy mildly heated the wound. Warming of wounds has been widely adopted to promote wound healing because the localized heat around the wound can increase the blood flow and oxygen tension in wounds, stimulate fibroblast proliferation, and reduce inflammation, which is beneficial for locally strengthening the metabolism around the wound and promoting wound healing. 2) The release of VEGF at an appropriate level facilitated vascularization. VEGF is released from this nanofiber system and stimulates multiple components of the angiogenic cascade. Capillary growth into the wound subsequently provides a conduit for nutrients and other mediators of the healing response as well as removal of metabolites, all of which are beneficial to wound healing. 3) The H_2_S generated from DA could regulate the immune microenvironment at the wound site and convert it from an inflammatory to an anti-inflammatory milieu. As is well known, H_2_S polarizes macrophages (M) from the pro-inflammatory M_1_-like phenotype toward the reparative M_2_-like phenotype, which have been found to promote tissue repair. To demonstrate our hypothesis, several evaluations on vascularization and immune regulation were performed.

**Fig. 6:**
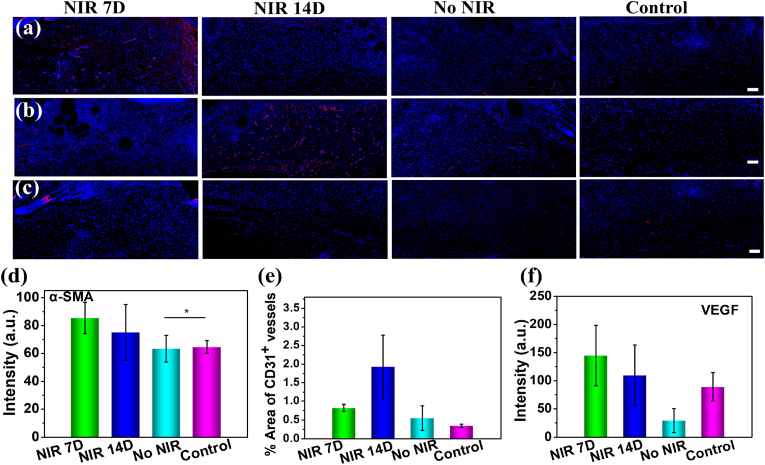
Immunohistochemistry staining and quantitative analysis evaluation of the MNFs@V–H@DA with 7 days NIR exposure, 14 days NIR exposure, no NIR exposure and control group on healing sites. α-SMA (a), CD 31 (b) and VEGF (c), the scale bars are 100 μm. Quantitative analysis evaluation of the MNFs@V–H@DA with various conditions responsive on α-SMA (d), CD 31 (e) and VEGF (f). Data are presented as mean ± standard deviation, n = 5, *p value < 0.05.

The staining of α-SMA (Fig. 6a), CD31 (Fig. 6b), and VEGF (Fig. 6c) were used to evaluate vascularization after different treatments. The quantified α-SMA was evaluated as a marker of the expression of smooth muscle cells, the results showed that MNFs@V–H@DA with 7-day NIR exposure produced a better α-SMA expression compared to that of the 14-day NIR exposure group, and which also was much higher compared to that without NIR (1.14-fold) or the control group (1.32-fold) (Fig. 6d). In addition, quantitative analysis evaluation of CD31 expression at wound sits indicated that the MNFs@V–H@DA group treated with MNFs@V–H@DA + 7 days of NIR demonstrated the much higher suppress local angiogenesis than that of the 14-day NIR exposure group, which was 2.35-fold lower compared to that with 14-day NIR irradiation and 1.40-fold greater than the control group (Fig. 6e). Furthermore, the VEGF expression after 7 days of MNFs@V–H@DA group with NIR exposure was much higher compared to other three groups, showing 4.95-fold and 1.32-fold enhancement compared to that without NIR exposure and the control group, more importantly, which also slightly higher compared to that after 14-day NIR irradiation (**Figure f),** but VEGF expression was still uniform, showing no aggregation phenomenon. These results indicated that the 7-day NIR group also showed better therapeutic effects compared to the 14-day NIR-treated group and no NIR and control groups, which suggested that MNFs@V–H@DA with 7-day NIR exposure could remodel microenvironmet for good new tissue formation.

**Fig. 7:**
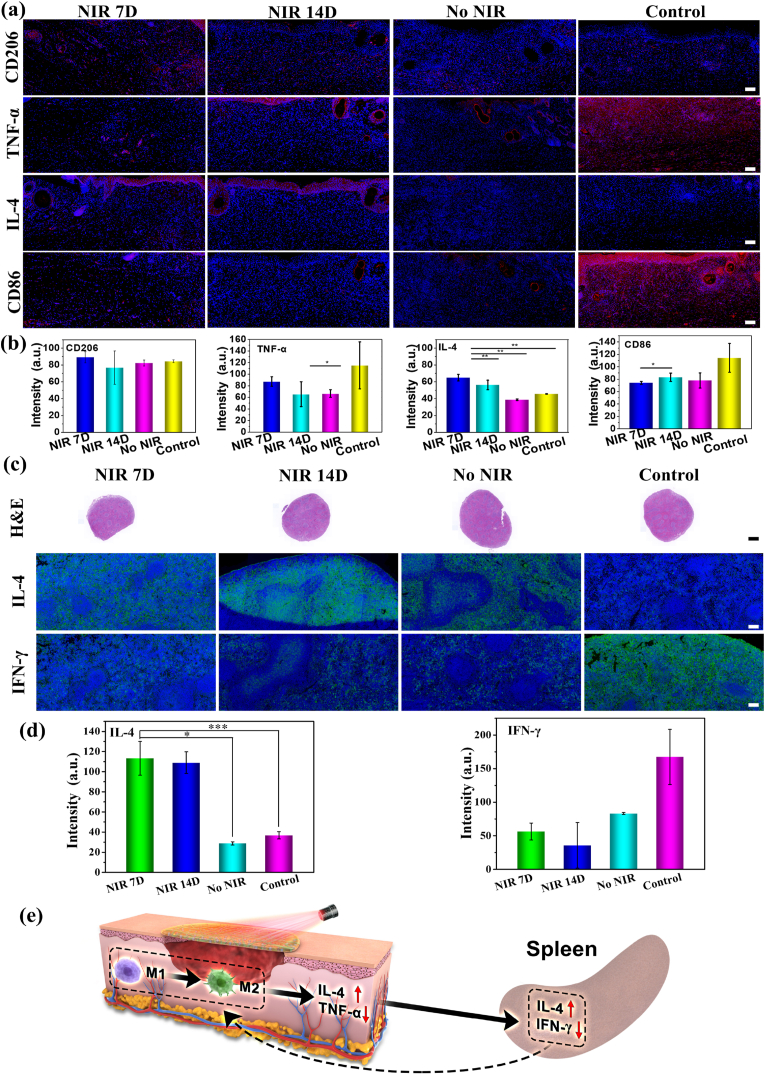
The immunomodulatory effects of MNFs@V–H@DA with NIR. Immunohistochemistry staining of the MNFs@V–H@DA on the healing sites with 7 days NIR exposure, 14 days NIR exposure, no NIR exposure and control group on healing sites.(a) of CD206, TNF-α, IL-4 and CD86 the bars were 100 μm. The quantitative analysis evaluation of CD206 (b), TNF-α (c), IL-4 (d) and CD86 (e). Immunohistochemistry staining evaluation (f) in spleen of HE, IL-4, IFN-γon the MNFs@V–H@DA, the bar of HE was 1 mm, the bars of IL-4, IFN-γ were 100 μm. The quantitative analysis immunohistochemistry staining evaluation in spleen of IL-4 (g), IFN-γ (h). The relevant immune mechanism analysis with NIR condition (i). Data are presented as mean ± standard deviation, n = 3, *p value < 0.05, **p value < 0.01, ***p value < 0.001.

To understand the immunomodulatory effects of MNFs@V–H@DA with NIR laser irradiation, immunohistochemical staining of IL-4, TNF-α, CD206, and CD86 was performed (Fig. 7a). These results indicated that CD206 and IL-4 expression in the MNFs@V–H@DA + 7-day NIR irradiation group were higher compared to that in the 14-day NIR, no NIR irradiation, and the control groups. Meanwhile, expression of CD86 was lower in the MNFs@V–H@DA + 7-day NIR group compared to that in the 14-day NIR, no NIR, and control groups. The expression of TNF-α in the MNFs@V–H@DA + 7-day NIR group was also lower than that in the control groups. In addition, quantitative analysis (Fig. 7b) revealed that the nanofiber system-treated groups showed a significant increase in conversion of the Macrophages from the pro-inflammatory M_1_-like phenotype toward the reparative M_2_-like phenotype compared to the control group due to the anti-inflammatory effect of H_2_S. In particular, MNFs@V–H@DA + 7-day NIR laser irradiation resulted in a 1.54-fold reduction in M_1_ and a 1.702-fold increase in M_2_ compared to the control group. Additionally, the expressions of pro-inflammatory factors (such as TNF-α, 1.32-fold, and anti-inflammatory factors (such as IL-4, 1.42 fold) were also decreased and increased, respectively, after treatment with MNFs@V–H@DA + 7-day NIR laser irradiation.

The results of IL-4 and IFN-γ immunohistochemical staining and H&E staining (Fig. 7c) in spleen tissue also indicated that MNFs@V–H@DA remodeled the immune microenvironment at the wound site by increasing M_2_ IL-4 and decreasing M_1_. Because the spleen is the main peripheral immune regulatory organ in the immune regulatory system and mainly secretes immune regulatory factors, we found that our treatment promoted the secretion of the anti-inflammatory factor IL-4 from the spleen and reduced secretion of the pro-inflammatory factor IFN-γ (Fig. 7d). This remote immune regulation will realize the transformation from a pro-inflammatory to an anti-inflammatory immune microenvironment at the wound site through further feedback regulation, which is more conducive to wound healing.

Based on the above results, we found that the wound tissue reaction to MNFs@V–H@DA with NIR laser irradiation began with modulation of the M_0_ response manifested in the M_2_-biased polarization. An increase in the population of M_2_ has been recognized as a crucial event for promoting wound healing, and a delayed transition from the pro-inflammatory M_1_ to immunomodulatory M_2_ phenotypes leads to fibrosis and scarring reactions and inhibits the regeneration of skin tissue (Fig. 7e). Thus, we believe that the appropriate vascularization and anti-inflammatory effects induced by this strategy are highly beneficial for achieving scarless wound healing.

## 3 Conclusion

In summary, we developed a novel NIR-responsive nanofibrous hydrogel band-aid to load and release VEGF and DATS, which provided desirable conditions for repairing wounds without scarring. The excellent photothermal effect of MNFs@V–H@DA can better meet the complex needs for healing wounds in a wider variety of clinical applications compared to traditional strategy. A large number of MXene nanosheets and VEGF in this fibrous skeleton not only endowed the MNFs@V–H@DA with an excellent photothermal effect but also provided functional groups to meet the requirements of cellular growth and vascularization and thus improved wound healing. Importantly, hyperthmia induced by PTT could control VEGF release, which also effectively limited the excessive angiogenesis. Furthermore, the DATS in the hydrogel provided binding inflammatory cells and produced H_2_S, achieving an effective anti-inflammatory effect and greatly facilitating rapid wound healing without scarring. Thus, we believe that our newly prepared and multifunctional band-aid has great potential in the field of scarless wound healing.

## 4 Experimental section

### 4.1 Materials

The PLGA (MW = 140.000 KD) were purchased from Daigang Polymer (Jinan, China); dopamine (DA) and hyaluronic acid (HA) were obtained from Sigma-Aldrich. VEGF and DATS were obtained from Sigma-Aldrich. MXene nanosheets as photothermal agents were prepared using our previously reported method [14]. All other chemicals were purchased from Guangzhou Chemical Co. and used without further purification. MSCs cells were obtained according to our previous method [21]. The BALB/c mice were purchased and cultured in the Center for Experimental Animals at Xi'an Jiaotong University Health Science Center. The protocol for animal experiments was approved by the Animal Experimentation Ethics Committee of Xi'an Jiaotong University.

### 4.2 Fabrication of the MNFs@V–H@DA

Each mL of electrospinning composite solution contained 120 mg of polymer (PLGA), 10 mg of SiO_2_@ VEGF (with 1 μg VEGF), and 20 mg of the obtained MXene nanosheets using DMF and DC compound absorbent (V = 2:8). The M-NFs@V was obtained by the electrospinning process according to the previous method [14].

MNFs@V–H@DA was prepared according to the following steps: First, DA-HA conjugate was prepared according to reported method [43]. 0.1 g of dopamine-hyaluronic acid conjugate and 1 mg of DA were dissolved in 1 mL of aqueous solution to prepare hydrogel precursor solution. Then, the hydrogel precursor solution was homogeneously coated on M-NFs@V through the spin coating method. Network structures of nanofibrous structure and hydrogel were obtained through oxidized reaction.

The temperature sensitivity of nanofibers could open/close their own surface and provide negative factors from the nanofibers and hydrogels, which play a key role in controlling the release of VEGF and DA.

### 4.3 Characterization of MNFs@V–H@DA

The chemical morphology elements of M-NFs@V and MNFs@V–H@DA were imaged using SEM and mapping. The TEM image was used to observe the structure of MXene nanosheets and SO_2_ NPs.

### 4.4 Photothermal conversion of the MNFs@V–H@DA

The photothermal performance of MNFs@V and MNFs@V–H@DA exposed to NIR laser and temperature changes were recorded using an infrared thermal imager (Fluke TIS 20+). Briefly, the MNFs@V–H@DA with 0.5 mL ultrapure water was added in a 24-well plate followed by exposure to an 808 nm NIR laser at 0.33 W/cm^2^, 0.5 W/cm^2^, and 1 W/cm^2^, respectively. To measure their photothermal stability, the MNFs@V–H@DA was irradiated with an 808 nm laser for three cycles, and temperature changes during this process were also recorded.

### 4.5 Cell culture and evaluation

MSCs were used to test the cellular response cells, and MNFs@V–H@DA. MNFs@V were set as control groups. The cellular viability analysis and morphology between MSCs and the surface of MNFs@V–H@DA were tested using previous work [34].

### 4.6 In vivo wound healing in a full-thickness skin defect model

The *in vivo* wound healing efficacies of different treatments were evaluated by a full-thickness skin defect model (female BALB/c mice [20–30 g, 5-6-weeks old]. First, all mice were randomly divided into 4 groups. Three groups were treated with MNFs@V–H@DA and irradiated with NIR light (808 nm, 0.33 W/cm^2^) for 0, 7, and 14 days, respectively. The group without any treatment was set as the control group. All surgery procedures were performed under aseptic conditions. Subsequently, the mice were anesthetized using an intraperitoneal injection of 5% chloral hydrate solution (10 mL/kg of body weight), and they were shaved in the dorsal region between the tail and back. The full-thickness skin round wounds (∼8 mm diameter) were created by a punch. In the control group, Transparent Film Dressing Frame Style was added to the wounds of mice only in the control group. Each wound area was measured and analyzed on day 1, 4, 7, 10, and 14.

For the immunohistochemical study, on day 14, the skins containing wounds and major organs (heart, liver, spleen, lung, and kidney) were collected (diameter = 1 cm) and fixed in the tissue fixative, and H&E, Masson, and immunofluorescent staining (α-SMA, CD31, VEGF, CD206, CD86, IL4, TNF-α, and IFN-γ) were performed. All operations followed the manufacturer's instructions.

## 5 Statistical analysis

All results are presented as the mean ± standard deviation. Comparison between the groups was assessed through a one-way analysis of ANOVA. Statistical significance was considered as *P* < 0.05.

## Data availability statement

Data available in the article and the supplementary material as well as by reasonable request from the authors.

## CRediT authorship contribution statement

**Lin Jin:** Resources, Methodology, Writing – original draft, Writing – review & editing. **Xiaoqing Guo:** Methodology. **Di Gao:** Conceptualization. **Yan Liu:** Validation. **Jiahua Ni:** Validation. **Zhiming Zhang:** Methodology. **Yiqiao Huang:** Methodology. **Guibin Xu:** Investigation. **Zhe Yang:** Writing – review & editing. **Xingcai Zhang:** Writing – original draft, Writing – review & editing. **Xianhan Jiang:** Writing – review & editing.

## Declaration of competing interest

The authors declare no competing financial interest.
