# Supplementary material for: An NIR photothermal-responsive hybrid hydrogel for enhanced wound healing
Source: Bioact Mater. 2022 Mar 10;16:162–72. doi: 10.1016/j.bioactmat.2022.03.006 (PMC8965777; doi:10.1016/j.bioactmat.2022.03.006)
Supplement: Multimedia component 1 [file mmc1.doc]

**A NIR photothermal-responsive hybrid hydrogel for enhanced wound healing**

Lin Jin,^a, b^ Xiaoqing Guo,^c^ Di Gao,^c^ Yan Liu,^d^ Jiahua Ni,^d^ Zhiming Zhang,^a^ Yiqiao Huang,^a^ Guibin Xu,^a^ Zhe Yang,^*c, f^ Xingcai Zhang^*d, e^, Xianhan Jiang^*a^

^a^Department of Urology, The Fifth Affiliated Hospital of Guangzhou Medical University, Guangzhou, 510700，P. R. China

^b^International Joint Research Laboratory for Biomedical Nanomaterials of Henan, Zhoukou Normal University, Zhoukou 466001, P. R. China

^c^The Key Laboratory of Biomedical Information Engineering of Ministry of Education, School of Life Science and Technology, Xi’an Jiaotong University, Xi’an 710049, P. R. China

^d^School of Engineering and Applied Sciences, Harvard University, Cambridge, MA, 02138, USA

^e^School of Engineering, Massachusetts Institute of Technology, Cambridge, MA, 02139, USA

^f^Research Institute of Xi’an Jiaotong University, Hangzhou, Zhejiang 311200, China

* Corresponding authors: [yangzhe@xjtu.edu.cn](mailto:yangzhe@xjtu.edu.cn) (Zhe Yang); [xingcai@mit.edu](mailto:xingcai@mit.edu) (X. Zhang); [Jiangxianhanz@126.com](mailto:Jiangxianhanz@126.com) (Xianhan Jiang)


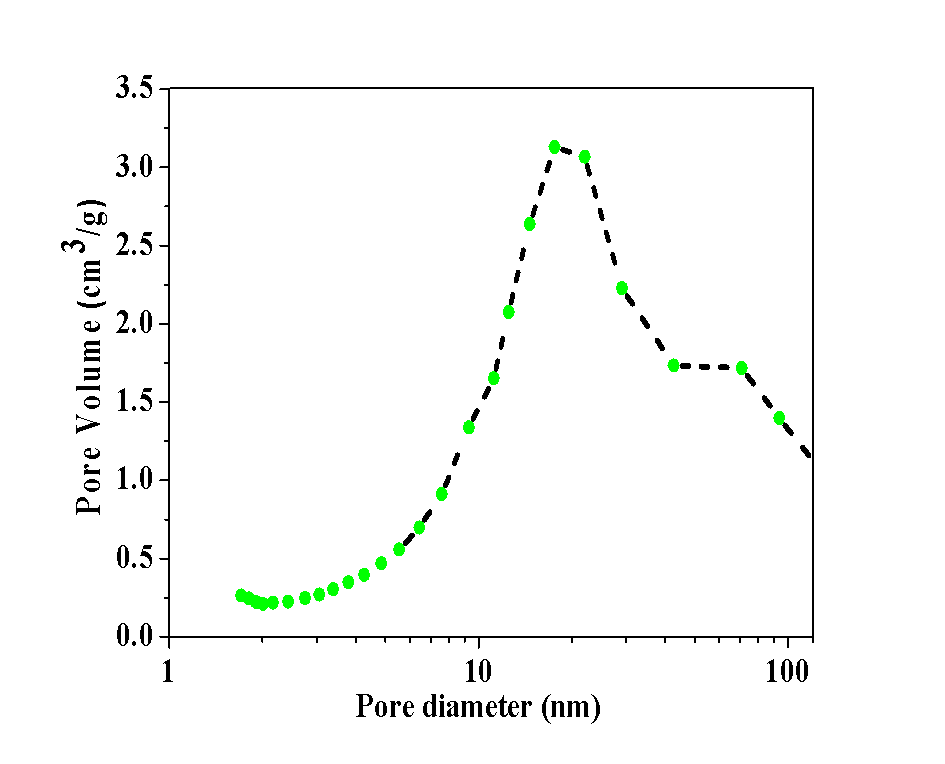


Figure S1 Pore diameter distribution of SiO_2_ NPs.


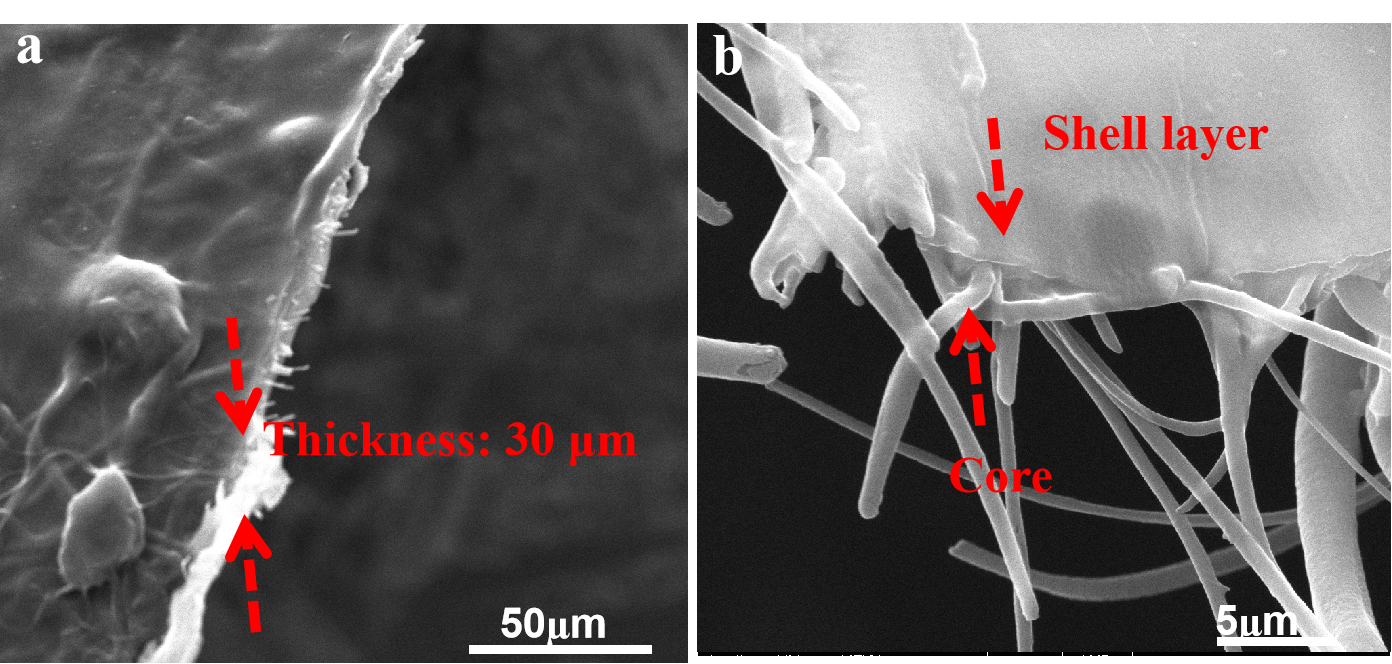


Figure S2 SEM images of MNFs@V-H@DA, (a) SEM image shows the thickness, (b) SEM image shows the shell layer and core layer.


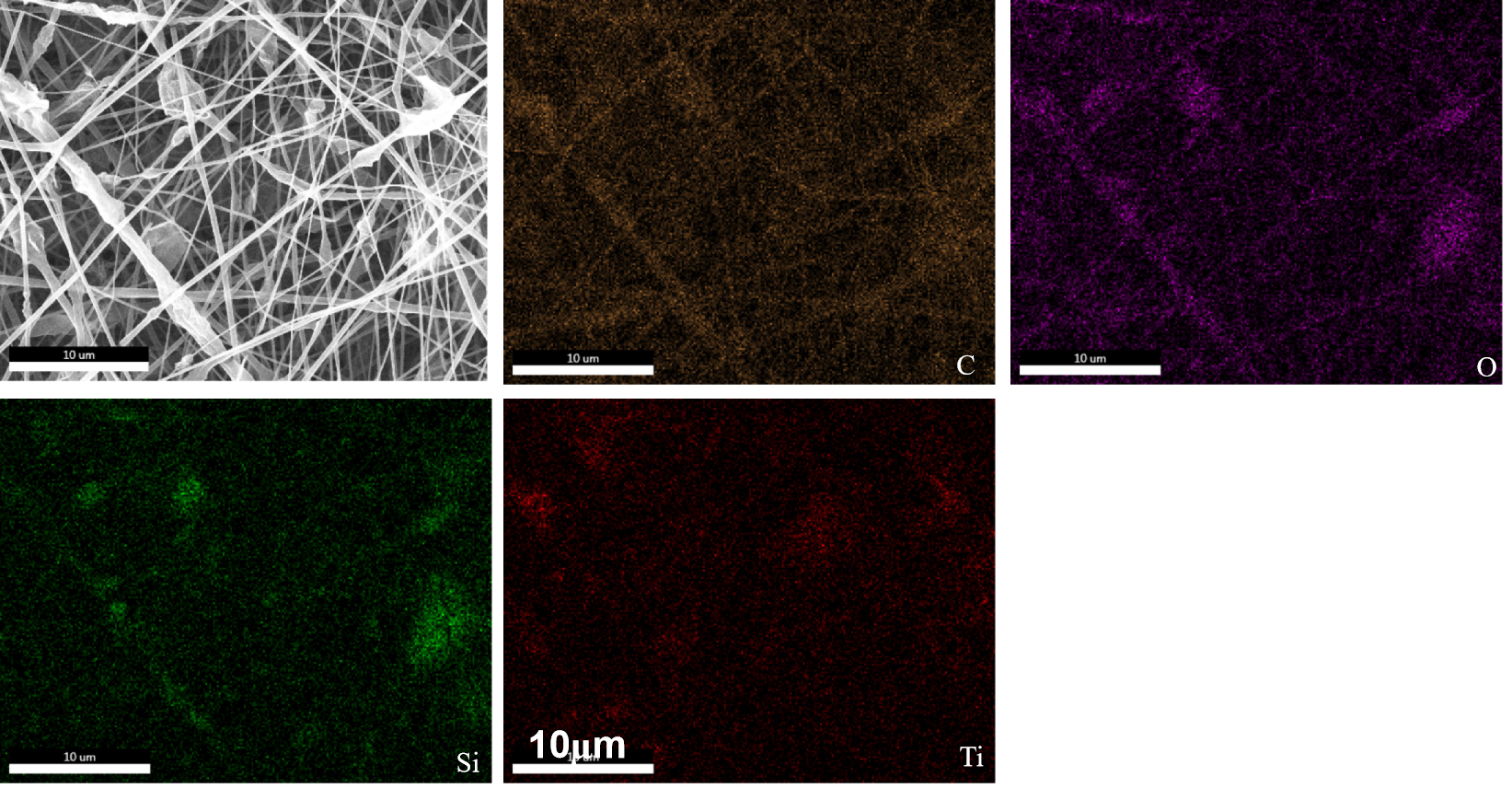


Figure S3 Mapping of MNFs@V.


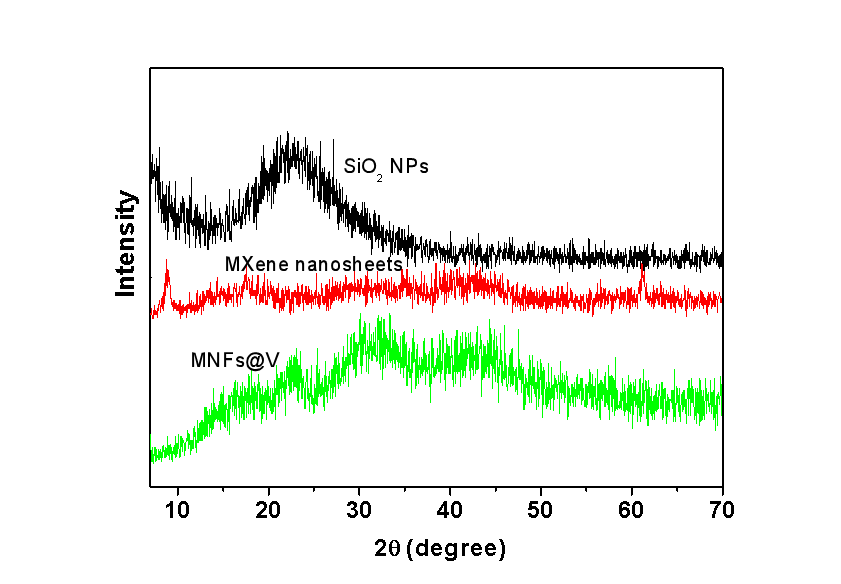


Figure S4 XRD pattern of SiO_2_ NPs, MXene nanosheets and MNFs@V.


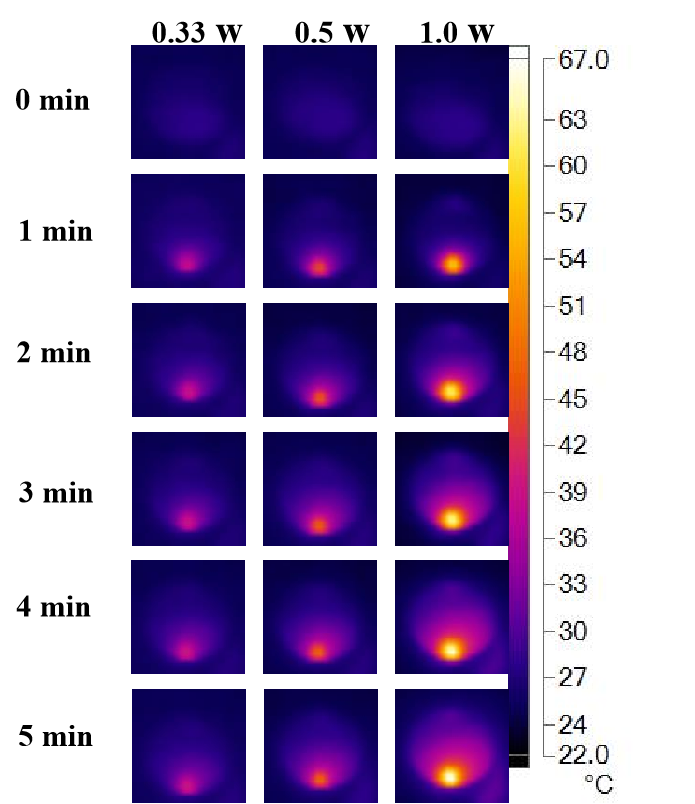


Figure S5 Thermal images of MNFs@V in the aqueous solution.


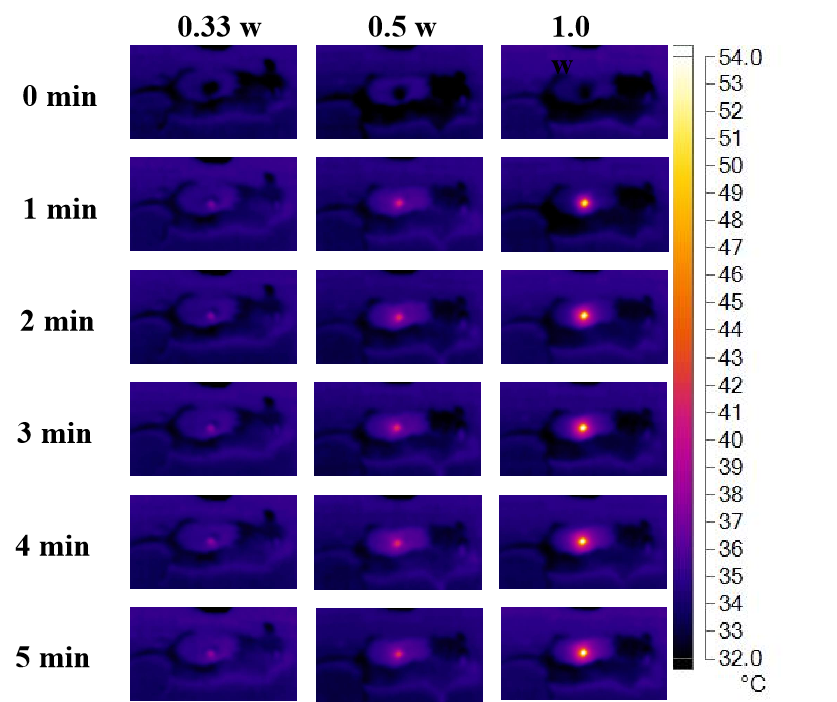


Figure S6 Thermal images of MNFs@V-H@DA on the wound sites.


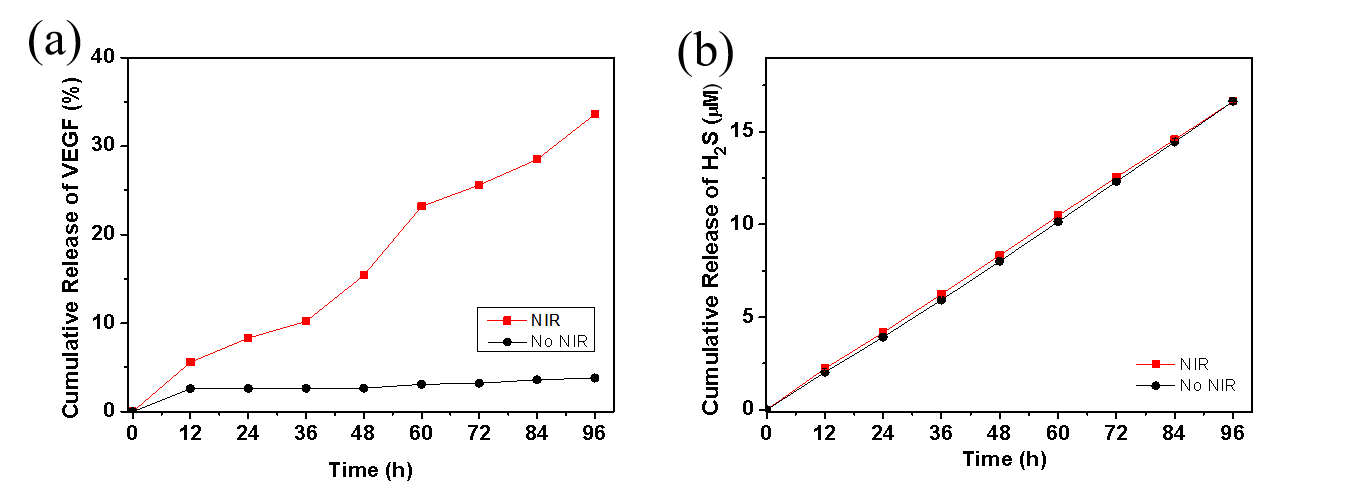


Figure S7 The cumulative release profiles of VEGF (a) and H_2_S with/without eight times NIR exposure every 12 h.


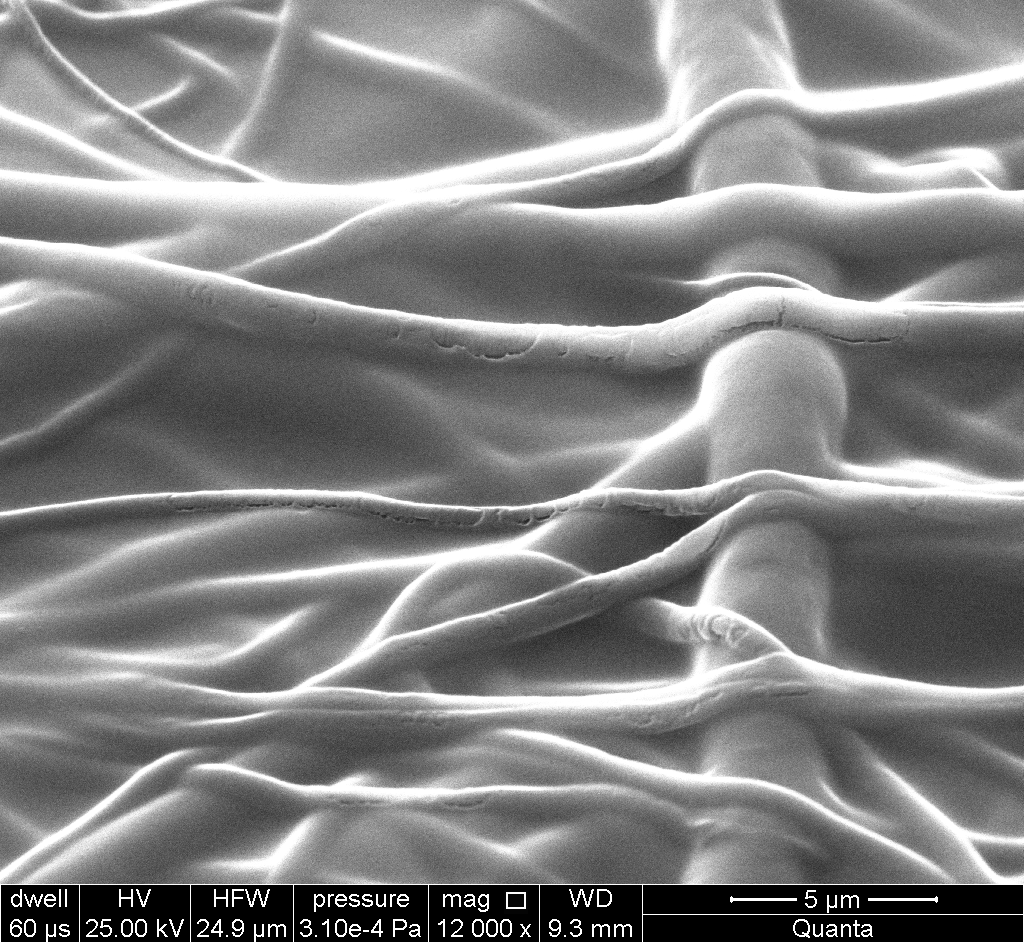


Figure S8 SEM image of MNFs@V-H@DA after NIR exposure.
